# Perceptually Easy Second-Language Phones Are Not Always Easy: The Role of Orthography and Phonology in Schwa Realization in Second-Language French

**DOI:** 10.1177/00238309241277995

**Published:** 2024-12-12

**Authors:** Elisabeth Heiszenberger, Eva Reinisch, Frederik Hartmann, Elizabeth Brown, Elissa Pustka

**Affiliations:** University of Vienna, Austria; Austrian Academy of Sciences, Austria; University of North Texas, USA; University of Vienna, Austria

**Keywords:** Orthography, phonology, French schwa, L2 French, imitation, reading

## Abstract

Encoding and establishing a new second-language (L2) phonological category is notoriously difficult. This is particularly true for phonological contrasts that do not exist in the learners’ native language (L1). Phonological categories that also exist in the L1 do not seem to pose any problems. However, foreign-language learners are not only presented with oral input. Instructed L2 learning often involves heavy reliance on written forms of the target language. The present study investigates the contribution of orthography to the quality of phonolexical encoding by examining the acoustics of French schwa by Austrian German learners—a perceptually and articulatorily easy L2 phone with incongruent grapheme-phoneme correspondences between the L1 and L2. We compared production patterns in an auditory word-repetition task (without orthographic input) with those in a word-reading task. We analyzed the formant values (F1, F2, F3) of the schwa realizations of two groups of Austrian high-school students who had been learning French for 1 and 6 years. The results show that production patterns are more likely to be affected by L1 grapheme-to-phoneme correspondences when orthographic input is present. However, orthography does not appear to play the dominant role, as L2 development patterns are strongly determined by both the speaker and especially the lexical item, suggesting a highly complex interaction of multiple internal and external factors in the establishment of L2 phonological categories beyond orthography and phonology.

## 1 Introduction

It is well known that, at the outset of foreign-language (L2) acquisition, learners can have considerable difficulties perceiving and producing non-native phonological contrasts (e.g., Cutler & Broersma, 2005; Escudero & Wanrooij, 2010; Flege et al., 1997; Levy & Law, 2010; Llompart & Reinisch, 2019a). Native speakers of German or Dutch, for instance, have been shown to have difficulties perceiving and producing the contrast between English /ε/ and /æ/ (e.g., *bet* [bεt] vs. *bat* [bæt]). They tend to map both categories onto the acoustically and articulatorily closest native (L1) category, which for both phonemes is /ε/, since /æ/ exists neither in German nor Dutch (e.g., Cutler & Broersma, 2005; Eger & Reinisch, 2019a, 2019b; Escudero et al., 2008; Llompart & Reinisch, 2017, 2019a, 2019b, 2020). The differentiation between phones with contrasts that also exist in the L1, such as English /i/-/ɪ/ (e.g., *sheep* [ʃip] vs. *ship* [ʃɪp]), on the other hand, does not appear to pose any problems (e.g., Broersma, 2005; Llompart & Reinisch, 2018; Weber & Cutler, 2004). Findings such as these are reflected in current models of L2 phonological development (Chang, 2019), for instance, the *(Revised) Speech Learning Model* (SLM/r-SLM; Flege, 1995; Flege & Bohn, 2021), the L2-*Perception Assimilation Model* (PAM-L2; Best & Tyler, 2007), or the *Second Language Linguistic Perception Model* (L2LP; van Leussen & Escudero, 2015). They assume that the degree of difficulty that learners experience in establishing new L2 phonological contrasts mainly depends on the phonetic proximity of a given L2 phone to the phonetically closest phone(s) in the L1. While phones that are phonetically almost identical between the L1 and L2 are easy to learn (since the L1 phone can also be used for the L2; the /i/-/ɪ/ example above), L2 phones that are similar but not too close are the hardest (i.e., the /ε/-/æ/ above^1^; Flege, 1987).

An additional obstacle for the establishment of an L2 phonemic inventory is that learners not only have to improve perceptual abilities so that they can accurately distinguish two L2 phonemes, but they also need to associate them with the correct L2 words. This process is commonly termed *phonolexical encoding* (Cook & Gor, 2015; Cook et al., 2016; Gor et al., 2021; Lancaster & Gor, 2016; Llompart, 2021b). Perceptually difficult L2 categories in particular may hinder establishing robust representations for L2 words. This is because the phonetic details of the individual phonemes making up the new L2 words are only “fuzzily” encoded into the representations of words in their L2 lexicon. For example, words including /æ/ appear to be “fuzzy” in terms of this vowel for German learners of English (e.g., Llompart, 2021a, 2021b; Llompart & Reinisch, 2017, 2019b). Most evidence for the *fuzzy lexical representation hypothesis* (Gor et al., 2021) comes from lexical decision tasks in which participants are asked to decide whether a given (non-)word is a word or not. For instance, Llompart (2021b) has shown in a lexical decision task, which included real words and nonwords created by substituting the L2 segment of interest by a confounding phoneme (e.g., *l[æ]*mon*, *dr[ε]*gon*), that German learners of English were able to more accurately perceive the substitution of /ε/ by /æ/ than when /æ/ was substituted by /ε/. This was even the case for highly proficient learners who were already able to distinguish the two vowels in a phonetic discrimination task. Such findings suggest that an increase in input frequency does not automatically lead to accurate phonolexical encoding, and imprecise lexical representations of particular words seem to remain even at high proficiency levels (e.g., Amengual, 2016; Darcy et al., 2013; Díaz et al., 2012; Gor et al., 2021; Llompart, 2021b; Simonchyk & Darcy, 2018). Llompart (2021a) has found that, especially for /æ/-nonwords, that is, words involving an unfamiliar L2 category, the quality of phonolexical encoding depends on phonological neighborhood and lexical frequency. The latter has been shown to lead to more accurate rejection patterns for /æ/-nonwords. According to Llompart (2021a), the fact that less frequent /æ/-nonwords are more likely to be rejected than highly frequent ones may result from exposure to error-prone L2 input. In the case of frequently used words, such as *thank*, learners may have been exposed to the mispronunciation *th[ε]nk more often, and therefore accept it as correct, than in the case of less frequently used words such as *habit*. It has therefore been argued that phonolexical representations of difficult L2 categories are modulated by the learner’s vocabulary size and exposure to native and non-native input, while the phonolexical encoding of easy L2 categories is instead related to acoustic properties, that is, phonetic proximity between L1 and L2 phonemes.

However, phonetic proximity between the L1 and L2 appears not to always be predictive of the difficulties that certain L2 phonemes or phones may cause. A case in point here is French “schwa” in words like *semaine* ([s[mεn] “week”] when produced by native speakers of Austrian German (Kamerhuber et al., 2020; Pustka et al., 2018). French “schwa” is acoustically and articulatorily close to the French full-front rounded vowels [œ] and [ø], and when the regional variation of the speakers is controlled for, the quality of French “schwa” is not more variable than its full-vowel neighbors (Bürki et al., 2008; Fougeron et al., 2007). Given that German also has front rounded vowels [œ] and [øː] (e.g., *Löffel* [ˈlœfl̩] “spoon,” *Flöte* [ˈfløːtə] “flute”), L2 learning models predict that Austrian German learners of French map French “schwa” onto these German counterparts in both perception and production. In other words, with regard to phonetic proximity, Austrian German learners could be expected to produce French schwa as tokens of [œ] or [øː], which in turn should result in productions close to the expected target for French schwa. However, Pustka et al. (2018) demonstrated in a reading task that this was not the case. Native speakers of Austrian German produced L2^2^ French schwa in initial syllables of polysyllabic words more fronted than expected in 39% of cases, that is, similar to the vowels [e] and [ε]. Since these vowels also exist in both French and German (Detey et al., 2016; Krech et al., 2009), the mere fact that learners are trying to keep their production of French schwa distinct from all other vowels of their L1 or L2 cannot explain this production pattern.

However, there are other possible explanations for the substitution of French schwa in initial syllables with vowels close to [e] and [ε] by Austrian German learners of French: First, while Standard German does have a reduced centralized vowel that could be considered schwa in unstressed syllables (e.g., *gemacht* [ɡəˈmaxt] “made”; Krech et al., 2009), this is not the case in Austria. This is because in Middle Bavarian varieties of German, which include most Austrian German varieties, this vowel is typically not reduced to a central vowel but produced as either a (short) full-front unrounded vowel (e.g., *gemacht* [ɡe̞ˈmaxt] “made”) or, especially in spontaneous speech, is deleted in initial syllables (e.g., *gemacht* [ɡˈmaxt] “made”; Hahn & Siebenhaar, 2017; Krech et al., 2009; Moosmüller et al., 2015). Second, the typical orthographic representation of schwa in French (and German) is <e>—with some exceptions in French such as in *Monsieur* [məsjø] “Mister” or *faisait* [fəzε] “made.” Substitution by vowels close to [e] and [ε] could therefore also be explained by interferences of L1 grapheme-phoneme correspondences.

The present study focuses primarily on the contribution of orthography to the quality of phonolexical encoding by examining the production of French schwa by Austrian German learners. Specifically, we assessed the role of congruent versus incongruent grapheme-phoneme correspondences between the L1 and L2 and compared schwa productions in a word-repetition task (where no orthographic input was available) with a word-reading task. We analyzed the values of the first three formants (F1, F2, F3) of French schwa produced by two groups of Austrian high-school students who had been learning French for either 1 or 6 years. French words were chosen so that schwa was either represented by the letter <e> or by other letter combinations such as <on> or <ai> (see above). Word repetition after a native French model speaker versus word reading was used to address L2 speech production with and without the immediate influence of orthography. We compared the production of schwa between learner groups (beginner vs. intermediate), spelling conditions (<e> vs. letter combinations), and tasks (orthography absent vs. present). Moreover, we compared the first three formant values of schwa to the first three formant values of French and Austrian German full vowels [œ], [øː], [e], and [ε], as well as of German unstressed <e> in initial syllables, which in Standard German would be expected to be pronounced as schwa (see above). These reference vowels were recorded from a subset of the Austrian learners in a reading task since they were not expected to vary much between conditions. This comparison will be termed the “reference condition.”

Due to the situation in Austrian German outlined above, the acoustic analysis of French schwa in Austrian learners of French provides a unique opportunity to investigate the interaction between phonetic proximity and orthography in L2 phonolexical encoding. The findings could extend our theoretical knowledge of the sources of L2 phonetic learning and the mapping between them and could have wider implications for language teaching, particularly in terms of emphasizing the role of orthography in learning to produce and encode L2 phones accurately. Before formulating the specific hypotheses of our study, we will provide the background information on the effects of orthography on L2 phonology, on which our hypotheses are based.

### 1.1 L1 influence on L2 word imitation vs. word reading

Taking perceptual abilities as an indicator of how robustly L2 phonological categories are encoded and differentiated from one another, imitation tasks are regularly used to assess phonological representations. Empirical evidence suggests that imitation abilities are constrained by L1 and L2 phonological categories (e.g., Flege & Eefting, 1988; Jia et al., 2006; Llompart & Reinisch, 2018; Schouten, 1977). Specifically, there is a closer match between imitation patterns and the perceptual categorization in the perception task than between imitation patterns and the acoustic properties of the acoustic input. For instance, Llompart and Reinisch (2018) tested German native speakers’ perception and imitation of two English phonological contrasts: /i/-/ɪ/ and /ε/-/æ/, which differed in the degree of perceptual difficulty. When asked to perceptually differentiate these contrasts, as predicted by current L2 phonological development models (e.g., Best & Tyler, 2007; Flege, 1995; Flege & Bohn, 2021), learners differentiated the /i/-/ɪ/ contrast, which is shared by the L1 and the L2, more accurately than the non-native /ε/-/æ/ contrast.

However, acoustic properties of imitated tokens do not always match with how speakers would realize the target categories in production tasks without an auditory model, for example, in reading tasks (Hao & de Jong, 2016; Llompart & Reinisch, 2018; Rojczyk, 2013; Rojczyk et al., 2013). For instance, Hao and de Jong (2016) showed that Mandarin native speakers rated Mandarin tones produced by English native speakers as more target-like when imitated than when read aloud by the same speakers. The authors consequently suggest that phonological categorization is bypassed during imitation, which in turn allows learners to accurately imitate difficult L2 phones for which they do not have native-like representations. In other words, learners are able to imitate phonetic details that are not phonologically relevant for them. Llompart and Reinisch (2018) also demonstrated that imitation patterns were not consistently associated with production patterns of the same phone in a reading task. However, in contrast to Hao and de Jong (2016), they argue that this might be partly due to the fact that phonetic discrimination does not automatically lead to an accurate phonolexical encoding of difficult L2 contrasts to L2 words. In other words, even though learners are able to distinguish between L2 contrasts in tasks which focus on phonetic abilities—such as imitation tasks—there may be a lack of phonetic detail in L2 lexical representations, that is, phonetic details are “fuzzily” encoded into these representations (see above). However, regardless of the underlying cause for a more faithful production of L2 phones in imitation tasks than in reading tasks, the latter differ per se by providing access to an additional type of information: orthography.

### 1.2 The role of orthography in learning L2 phonological systems

Research on L2 learning has reported that orthography is one of the contributing factors to learners’ success at establishing a lexical distinction of L2 words, including difficult L2 phonological categories. This is because instructed L2 learning often involves a heavy reliance on written forms of the target language (Bassetti et al., 2015). It has been shown that orthography can affect learners’ ability to distinguish between difficult L2 phone contrasts in perception and production (e.g., Bassetti, 2006, 2023; Escudero et al., 2008; Escudero et al., 2014; Hayes-Harb & Cheng, 2016; Hayes-Harb et al., 2010; Showalter, 2018; Showalter & Hayes-Harb, 2013). Several findings suggest that the availability of two different graphemes for two perceptually difficult L2 phones may serve as a cue signaling phonological contrast (e.g., Brown, 1998; Eckman, 2004; Escudero et al., 2008; Escudero et al., 2014; Showalter & Hayes-Harb, 2013; Steele, 2005). For instance, Escudero et al. (2008) have shown that native speakers of Dutch who learned novel English words together with their spellings perceptually distinguished between /ε/ and /æ/ (a difficult L2 contrast) better than learners who were presented with only spoken forms during learning. Relatedly, Showalter and Hayes-Harb (2013) also reported a facilitative effect of unfamiliar diacritics in the written input in the acquisition of novel L2 lexical tone contrasts. In that study, native speakers of English were taught a set of Mandarin nonwords contrasted by lexical tone. Half of the learners saw Romanized written forms of Pinyin with tone marks (e.g., <fiān>), and the other half saw Pinyin without tone marks (e.g., <fian>). The group provided with tone marks during training more accurately matched the nonwords to visual referents. Orthographic input and visual marking of L2 phonetic contrasts may therefore facilitate the distinction between perceptually difficult L2 phonemes.

Despite strong evidence for facilitative effects of orthography on L2 learning, several findings contradict this view. While only a small number of studies reported an absence of orthographic effects (e.g., Showalter, 2012; Simon et al., 2010), several studies demonstrated how orthography may provide misleading information, and hence negatively affect L2 phone learning (e.g., Bassetti, 2007, 2017, 2023; Hayes-Harb et al., 2010; Hayes-Harb & Cheng, 2016; Mathieu, 2016; and references therein). Specifically, learners appear to incorrectly map L2 phones to a given L1 category when they are represented by the same grapheme (e.g., Bassetti, 2017; Hayes-Harb et al., 2010; Hayes-Harb & Cheng, 2016; Mathieu, 2016; Piske et al., 2002; Zampini, 1994;). For instance, Hayes-Harb et al. (2010) taught native speakers of English sets of nonwords referring to visual referents, with participants being assigned to one of three groups: one saw a non-informative mask <XXXX> below the pictures, another saw written forms consistent with English grapheme-phoneme correspondences (e.g., <fasha> for [faʃə]), and one saw written forms that were inconsistent with English grapheme-phoneme mappings (e.g., <faza> for [faʃə]). In a test phase, the group that had encountered incongruent grapheme-phoneme mappings was more likely than the other two groups to accept (mis)pronunciations like [fazə] as the correct form for the pictures originally referred to as [faʃə], likely because the English grapheme <*z*> typically represents the phone [z]. Along the same lines, Mathieu (2016) and Hayes-Harb and Cheng (2016) tested the effects of varying grapheme familiarity in novel L2 lexical items. They found that L2 learners produce non-native phone contrasts more accurately when exposed to an entirely unfamiliar script during the word learning phase than when represented with incongruent grapheme-phoneme correspondences between the L1 and L2, reinforcing the argument that incongruencies between L1 and L2 grapheme-phoneme correspondence negatively affect L2 learning.

Despite the focus on the investigation of novel words or pseudowords, there are also a few findings that show negative effects of orthography in experienced L2 learners producing real words (Bassetti, 2017; Bassetti & Atkinson, 2015). Bassetti and Atkinson (2015), for instance, showed orthographic effects on Italian learners’ pronunciation of English words of the type *lamb* /læm/, where learners tended to pronounce the [b] as suggested by the written form. Crucially, across different tasks, speech production was more affected by orthography in reading than in an immediate repetition task. Along the same lines, Bassetti (2017) found that Italian learners of English distinguished between singleton and geminate consonants in production depending on the L2 orthographic form analogous to the coding of this contrast in their L1. This was despite the fact that in English, no such distinction exists. Interestingly, in this case, the orthographic influence was found in a reading task as well as in a delayed repetition task without direct orthographic input, suggesting that not only L2 speech production but also L2 phonolexical representations might be influenced by orthography.

### 1.3 The present study: summary and predictions

This study aimed to address the extent to which orthography affects L2 speech production patterns of a perceptually easy L2 phone with incongruent grapheme-phoneme correspondences between the L1 and L2. For this purpose, we examined imitation and production patterns of French schwa by Austrian German high-school students learning French at school (31 beginners and 19 intermediate learners). Specifically, we measured the first three formants (F1, F2, F3) of learners’ schwa productions, as they (indirectly) provide information about tongue position and lip rounding: while F1 reflects tongue height, F2 is primarily related to the tongue position on the horizontal plane (front to back). F3 reflects lip rounding, among other factors.

The students performed a word-repetition task following a native French model speaker, without seeing the words’ orthography, and a word-reading task. In the imitation task, the students heard a recording of a Parisian newscaster reading aloud a list of French words containing words with schwa in initial syllable (e.g., *semaine* [səˈmεn] “week”),^3^ among others. Stimuli were chosen to be either spelled such that schwa was represented by the letter <e> or by other letter combinations such as <on> or <ai> . The students were then asked to repeat each word as quickly as possible. In the word-reading task, the participants had to read aloud a list of written words in French including inter alia the same words as in the imitation task. Results from these tasks served to investigate differences in the French schwa productions between two tasks, in which orthography was either present or absent, and between different levels of language proficiency. Since the aim of the present study was to examine whether French schwa productions were affected either by the phonetic proximity of L1 to L2 phones or by incongruences between L1 and L2 grapheme-phoneme correspondences, the F1, F2, and F3 of schwa were compared to those of the closest French and Austrian German full vowels [e], [eː], [ε], [œ], and [øː]. Productions for this reference condition were obtained from a subset of participants and involved reading aloud lists of written words in German and French.

With respect to the learners’ imitation patterns of schwa realizations, diverging predictions can be made about the relationship between orthography and L2 speech production, depending on whether imitation abilities are constrained by phonolexical representations or not (Flege & Eefting, 1988; Hao & de Jong, 2016; Jia et al., 2006; Llompart & Reinisch, 2018; Rojczyk, 2013; Rojczyk et al., 2013; Schouten, 1977). If phonolexical encoding was bypassed in imitation, then a priori no link between orthography and imitation patterns of schwa is expected. Based on the fact that French schwa, which is acoustically and articulatorily similar to the rounded full vowels [œ] or [ø] (e.g., Bürki et al., 2008), has easily identifiable counterparts in German [œ] and [øː], learners should not have difficulties in imitating schwa accurately, regardless of its spelling. By contrast, if imitation patterns were related to phonolexical representations, diverging predictions can be made about acoustic characteristics of imitation patterns of schwa, depending on the role attributed to orthography in phonolexical representations. If phonolexical representations are influenced by orthography, it is in principle expected that learners would face other difficulties with imitating schwa corresponding to French <e> than with imitating schwa corresponding to <ai> or <on> . This means that in the case of <e>, they would realize schwa more fronted and less rounded, that is, like German [e]/[eː] or [ε] and not like [œ] and [øː]. If, on the contrary, orthography does not play a role in phonolexical representations, but L1 phonological categories are important regardless of their spelling, imitation patterns of schwa by the Austrian students should be found to be inaccurate, that is, close to the production of German [e] in unstressed position (see Section 1) and no difference between instances of schwa corresponding to <e> and schwa corresponding to <ai> or <on> would be expected.

Concerning the learners’ production patterns of schwa realizations in the reading task, it could be expected that acoustic characteristics of schwa imitation patterns would differ from those of schwa reading patterns. First, this is because previous studies have shown that L2 learners had less difficulty imitating than producing L2 sounds without an auditory model (Hao & de Jong, 2016; Rojczyk, 2013; Rojczyk et al., 2013). Second, it is likely that in the reading task, there is a higher impact of orthography on production patterns than in the imitation task due to the presence of the orthographic input. Specifically, if production patterns of French schwa were indeed related to the presence of orthographic forms at the moment of speech production, we would expect to find that schwa is pronounced more fronted and less rounded, that is, similar to German [e]/[eː] or [ε], than in the word-repetition task. This should be observable especially for schwa corresponding to <e>, where interferences are expected to be more common. However, it is also possible that the different spelling, such as <on> or <ai>, causes further difficulties and production patterns become fuzzy.

## 2 Methods

### 2.1 Participants

To investigate the potential influence of orthography on the imitation and production of French schwa, speech production data were collected from two groups of native Austrian German-speaking high-school students in Vienna, Austria, who differed in terms of their French proficiency as indicated by their duration of learning: 31 students were in their first year of French classes at school (18 female, 13 male, ages 12–13), and 19 students were in their sixth year (15 female, 4 male, ages 17–18). None of them had received any instruction in or extended exposure to French before starting to learn the language at school. All participants were students at the same Viennese high school (*Gymnasium*) and, at the time of testing, had been learning English as their first foreign language (L2) for 2 and 8 years, respectively. French was consequently their second foreign language (L3). In the case of bilingual students, English was their L3, and French their L4. However, since we did not expect any specific influence of English on the outcomes of the present study, for simplicity, we refer to French as the L2. Permission to conduct the recordings was granted by the headmaster of the school as well as the Vienna supervisory school authorities (*Stadtschulrat*). All students and their parents gave informed written consent to participate. The students were recorded as part of the larger research project *Pronunciation in Progress* (Pro^2^ F, https://pro2f.univie.ac.at, Heiszenberger et al., 2020; Kamerhuber et al., 2020).

For the reference condition, data were collected from 26 Austrian high-school students (16 female, 10 male). Inclusion and exclusion criteria were the same as above, except that participants in the reference condition were already 15–16 years old at the time of the recordings, and therefore in their third year of learning. This is due to the fact that the German word-reading task including words allowing acoustic analyses of the critical vowels [eː], [ε], [œ], and [øː], as well as of the realization of <e> in unstressed initial syllables corresponding to schwa in Standard German, was solely part of a third recording session within a longitudinal study. In the latter, only first-year students were recorded once every year. This means that all of them were also part of the main study. As five students had left the school at which recordings took place between the first and the third year of learning, data from only 26 students were collected for the reference condition.

### 2.2 Materials and procedure

The main study consisted of two tasks: a word-repetition task after a native speaker and a word-reading task. Materials and procedure for each task are described in detail in the following subsections. All participants completed the two tasks at school in one recording session that additionally included five other tasks to assess various other aspects of learning French as a foreign language. In the recording session, participants first completed the word-repetition task. This was to avoid potential influences from word reading on imitation. In addition, to avoid potential influences from imitation onto the reading aloud of the same words, the reading aloud of the word list did not immediately follow the word-repetition task. Only after all tasks in French were completed were participants asked to read aloud a German text. All second-language tasks were conducted entirely in French. The word-repetition and word-reading tasks were conducted by an Austrian German native speaker who was a trained teacher of French (i.e., speaking French at a high proficiency level, matching C1 according to the Common European Framework of reference for languages levels; Council of Europe, 2020).

Participants were tested individually in a quiet room at the high school. Recordings were made using an H4n Pro portable 4 track recorder (24-bit 96 kHz WAV file). Participants wore an AKG C520 headset microphone placed at the lip-line. In both tasks to be analyzed here, the order of words was randomized. The same random order was used for all participants. Recordings were timed manually and controlled by the experimenter. If a word was not recorded properly due to noise, the word had to be repeated. In the case of mistakes in word repetition or reading, the participants had the opportunity to correct themselves. Each of the two tasks took approximately 4–5 minutes.

### 2.3 Word-repetition task

The word-repetition task served to assess the production of French schwa without seeing the words’ orthography. Learners were presented with 31 French words and expressions, recorded by a 58-year-old male Parisian newscaster. The words were presented one at a time through speakers at a comfortable listening level. After each word, learners were asked to repeat the word as quickly as possible. For the present study, we analyzed learners’ repetitions of eight words and expressions. They all contained schwa in the initial syllable: *petit-déjeuner* [pətideʒœne] “breakfast,” *chemise* [ʃəmiz] “shirt,” *sera* [səʁa] “(he or she) will be,” *semaine* [səmεn] “week,” *Monsieur* [məsjø] “Mister,” *faisait* [fəzε] “made,” *je sais pas* [ʒəsεpa]^4^ “I don’t know,” and *de temps en temps* [dətɑ͂zɑ͂tɑ͂] “from time to time.” The selected words and expressions were based on textbooks from the series *Bien fait!* used in French classes, to ensure that they were learned early and used frequently in the classroom (Luner & Berchotteau, 2009, 2010; Luner et al., 2008; for an overview, see also the work by Kamerhuber et al., 2020). Note that each word was spoken preceded by a pause, which ensured that the schwa had to be produced (for production rules of French schwa, see the study by Armstrong, 2001; Lyche, 2016). None of the schwas were therefore elided by the Parisian native speaker. To focus on how French orthography affects schwa realizations in initial syllables and avoids confounds from L1 phonological forms, French-German cognates such as *premier* [pʁəmje] “premier” (germ. Premiere [pʁe̞mˈjeːʁə]) were not included in the present analysis.

### 2.4 Word-reading task

A word-reading task assessed the learners’ production of French schwa without an auditory model. For the word-reading task, the participants were seated in front of a printed word list. They were informed that they could place the word list at a comfortable reading distance, and that they had to wait for a visual sign from the interviewer before reading the given words aloud. For the word-reading task, the same words and expressions were elicited as in the word-repetition task (see above). They were presented as part of a word list including 97 French words. Each word was preceded by a pause preventing a context for schwa deletion.

### 2.5 Reference condition

Since the purpose of the present study was to investigate whether speakers’ ability to imitate and produce a perceptually easy L2 sound is constrained by the phonetic proximity of L2 sounds to L1 sounds or by L1 grapheme-phoneme correspondences, we additionally analyzed the production of German and French vowels. Specifically, we analyzed the first three formants (F1, F2, F3) of the critical vowels [eː], [ε], [œ], and [øː] and of German <e> in initial syllables, which would be expected to be pronounced as schwa in Standard German, produced by a subgroup of the Austrian high-school students.

The reference condition consisted of two tasks: the reading aloud of a German and a French word list. As in the main study, all participants completed the two tasks in one recording session which also included five other tasks. Only after all tasks in French were completed (conducted entirely in French) were participants asked to read the German word list aloud. The procedures were identical to those in the main study.

For the analysis of the production of French vowels by Austrian high-school students, we used the reading aloud of the Phonologie du Français Contemporain (PFC) word list, which is also used within the recording protocol of the international research program (I)PFC (Durand et al., 2002; Racine et al., 2012), which analyzes the French pronunciation of L1 and L2 French speakers all over the globe. Materials for the French word-reading task were thus 94 French words chosen especially for the analysis of the phonemic system of L1 and L2 French speakers. For the present study, five words for each of the following critical sounds—[ε], [e], [œ], and [øː]—and five words for each of the following cardinal vowels—[i], [u], and [a]—were selected to be analyzed acoustically for each participant.

For the analysis of the production of German vowels by the Austrian high-school students, we investigated the reading aloud of a word list, including 46 German words. Again, five words for each of the following critical sounds—[ε], [e], [œ], [ø], and the pronunciation of <e> in initial syllables which would be expected to be pronounced as schwa in Standard German—and five words for each of the following cardinal vowels—[i], [u], and [a]—were selected to be analyzed acoustically for each participant.

### 2.6 Data analysis

For the acoustic analysis of the learners’ productions of French schwa, we first manually segmented each vowel and then examined the formant structure (F1 F3) using the software PRAAT (Boersma & Weenink, 2023). For the segmentation of vowels, we used standard phonetic criteria: boundaries were placed at the onset and offset of energy at F2 (Figueroa & Young, 2021; Turk et al., 2006). Extraction of the first three formant frequencies was conducted automatically using a PRAAT script created by Philip Hoole (LMU Munich) and Léa Courdès-Murphy (University of Vienna). The script extracted the median values (in Hertz) of the first three formants over the whole vowel. Subsequently, we plotted all formant frequencies in the F1 F2 space for each speaker individually to verify the results in terms of plausibility. In cases where the automatic extraction yielded obviously wrong results, corrections of formant frequencies were made manually.

To minimize inter-speaker variation due to physiological or anatomical differences, we normalized formant frequencies for each participant by applying the Lobanov procedure (Lobanov, 1971). The Lobanov-normalization is a speaker-intrinsic, formant intrinsic, and vowel-extrinsic method, which is considered to preserve phonemic variation best of all procedures (Adank et al., 2004). When using this method, the formant values are converted to z-scores. That is, a given formant frequency of the target vowel (e.g., F2 of our expected schwa production) is normalized by first subtracting the speaker’s mean formant frequency for F2 across all German and French vowels and then dividing it by the standard deviation for the formant frequencies of all vowels produced by that speaker. Since the Lobanov-method is vowel extrinsic, formant frequencies of more than one vowel for each speaker are required. Therefore, we also extracted the first three formant frequencies of the French and German cardinal vowels [a], [i], and [u], produced by each speaker (5 tokens per vowel per speaker). The formant frequencies were then compared to those of German and French critical vowels—[ε], [e], [œ], and [ø], as well as the pronunciation of <e> in initial syllables which would be expected to be pronounced as schwa in Standard German—produced by a subset of the Austrian students (see the Reference condition section). In the case of the word-repetition task, results were also compared to the auditory input, that is, normalized formant frequencies of French schwa realizations produced by a 58-year-old male Parisian newscaster. Formant frequencies were normalized in the same way as for the Austrian students. In total, we analyzed 747 tokens for French schwa in initial syllable: 378 in the imitation task and 369 in the reading task.

### 2.7 Model

To analyze the data, we used the programming interface STAN to design two Bayesian hierarchical regression models with multivariate normal outcomes for each formant. Model 1 was created to model the speakers’ realizations of schwa in the French examples, and Model 2 was used to model each speaker’s reference realization of French and German vowels other than schwa.^5^

The variables we model with our analysis are the learning stage (L) as a binary variable (0–beginner, 1–advanced), the task *reading* vs. *repetition* (*task*), the word in question (*word*), and the speaker (*speaker*). We can infer the influence of those variables on the three formant values (the outcome variables) and disentangle their effects.

The advantage of using hierarchical multivariate normal models is that the three formant values can be modeled jointly in the same model where at each level, the joint effects of the predictors on all three formants are inferred simultaneously. This makes the inferences more robust and models the effects on the formants more accurately than separate models for each formant could. This is because, in reality, the three formants are linked (i.e., all three are properties of a single sound and are not logically independent variables). Because of this, modeling the formants as linked in the analysis is more adequate and improves the model’s estimates. Equation 1 shows the model equation for Model 1 which models the realization of schwa in the learners’ French productions in the reading versus the repetition tasks.

(1) $$\begin{array}{c} \left[{F_{1}}_{i},{F_{2}}_{i},{F_{3}}_{i}]\sim MultiNormal([{\mu_{1}}_{i},{\mu_{2}}_{i},{\mu_{3}}_{i}],K\right)\\ {\mu_{1}}_{i}=\alpha_{1}+{{\beta_{1}}_{task}}_{i}+{{\gamma_{1}}_{word}}_{i}+{{\delta_{1}}_{speaker}}_{i}\\ +(\epsilon_{1}+{{\zeta_{1}}_{task}}_{i}+{{\eta_{1}}_{word}}_{i})L_{i}\\ {\mu_{2}}_{i}=\alpha_{2}+{{\beta_{2}}_{task}}_{i}+{{\gamma_{2}}_{word}}_{i}+{{\delta_{2}}_{speaker}}_{i}\\ +(\epsilon_{2}+{{\zeta_{2}}_{task}}_{i}+{{\eta_{2}}_{word}}_{i})L_{i}\\ {\mu_{3}}_{i}=\alpha_{3}+{{\beta_{3}}_{task}}_{i}+{{\gamma_{3}}_{word}}_{i}+{{\delta_{3}}_{speaker}}_{i}\\ +(\epsilon_{3}+{{\zeta_{3}}_{task}}_{i}+{{\eta_{3}}_{word}}_{i})L_{i}\\ \left[\alpha_{1},\ldots ,\alpha_{3},\epsilon_{1},\ldots ,\epsilon_{3}]\sim MultiNormal([{\bar{\mu }}_{1},\ldots ,{\bar{\mu }}_{6}],K_{b}\right)\\ \left[{{\beta_{1}}_{task}}_{i},\ldots ,{{\beta_{3}}_{task}}_{i},{{\zeta_{1}}_{task}}_{i},\ldots ,{{\zeta_{3}}_{task}}_{i}\sim MultiNormal([0,\ldots ,0]K_{t})\right]\\ \left[{{\gamma_{1}}_{word}}_{i},\ldots ,{{\gamma_{3}}_{word}}_{i},{{\eta_{1}}_{word}}_{i},\ldots ,{{\eta_{3}}_{word}}_{i}]\sim MultiNormal([0,\ldots ,0]K_{w}\right)\\ \left[{{\delta_{1}}_{speaker}}_{i},\ldots ,{{\delta_{3}}_{speaker}}_{i}]\sim MultiNormal([0,\ldots ,0]K_{s}\right) \end{array}$$

Partial model equation for the formant model of speakers’ schwa realizations. See Appendix for full equation.

At its core, the model is a linear mixed-effects model with varying slopes and varying intercepts. Compared to simple linear mixed-effects models, the architecture of the model is extended to better model the effects of the variables on the three formants. All three formants are modeled jointly as dependent variables as the outcome of a multivariate normal distribution.^6^ The model further contains the variables *task*, *word*, *speaker*, and *language proficiency* as predictors. All these predictors, along with their intercepts, are modeled as multilevel variables (i.e., as a varying slope-varying intercept mixed-effects model) jointly for all three formants. This has the advantage that the intercepts and slopes of one predictor can be jointly inferred at the same time in the same distribution. Doing this links the estimation of those predictors and makes them more accurate. Other than in Model 2 (see below), we decided not to use interaction effects between the variables to keep the model effects interpretable.

As discussed above, the model in Equation 1 uses multivariate normal outcome distribution in two places: the modeling of the outcome formants as well as the effects for the predictors for each formant. We can see in Equation 1 that in this model, the outcomes (dependent variables) F1, F2, and F3 are modeled as multivariate normal outcomes with means $\mu_{1}$ to $\mu_{3}$ and a covariance matrix K. Furthermore, each formant’s mean is inferred as a linear combination of an intercept α, and group-level intercepts β (for the task reading and repetition), γ (for word), and δ (for speaker). The effect of the predictor *language proficiency* is encoded in the term $(\epsilon +\zeta_{\mathrm{task}}+\eta_{\mathrm{word}})L$ where L is a binary variable with year one learners coded as 0 and year six learners coded as 1. The entire term therefore equals 0 when the datapoint at position i represents a beginner. Accordingly, ϵ is the base effect of proficiency while ζ and η are group-level differences in the proficiency effect of task and word.

To model the predictors as multivariate variables themselves, all slopes and intercepts for each formant equation are drawn from multivariate normal distributions with an individual covariance matrix per distribution. This means that at all levels of the model, the three formants are considered as linked via stacked multivariate normal distributions. For priors, we chose weakly informative priors with more regularizing priors on α and ϵ. Refer to the Appendix for the full model equation.

The second model is intended to infer each speaker’s formant values for individual vowels other than schwa to compare them to the schwa realizations. It builds on a similar principle; however, this time, it includes interaction effects. Equation 2 shows a model equation which includes the main terms.

(2) $$\begin{array}{c} \left[{F_{1}}_{i},{F_{2}}_{i},{F_{3}}_{i}]MultiNormal([{\mu_{1}}_{i},{\mu_{2}}_{i},{\mu_{3}}_{i}],K\right)\\ {\mu_{1}}_{i}=\alpha_{1}+{{\beta_{1}}_{phoneme}}_{i}+{{\gamma_{1}}_{language}}_{i}+{{\delta_{1}}_{speaker}}_{i}+{\zeta_{1}}_{{language}_{i},{phoneme}_{i}}\\ +{\eta_{1}}_{{language}_{i},{speaker}_{i}}+{\theta_{1}}_{{speaker}_{i},{phoneme}_{i}}+{\iota_{1}}_{{language}_{i},{speaker}_{i},{phoneme}_{i}}\\ {\mu_{2}}_{i}=\alpha_{2}+{{\beta_{2}}_{phoneme}}_{i}+{{\gamma_{2}}_{language}}_{i}+{{\delta_{2}}_{speaker}}_{i}+{\zeta_{2}}_{{language}_{i},{phoneme}_{i}}\\ +{\eta_{2}}_{{language}_{i},{speaker}_{i}}+{\theta_{2}}_{{speaker}_{i},{phoneme}_{i}}+{\iota_{2}}_{{language}_{i},{speaker}_{i},{phoneme}_{i}}\\ {\mu_{3}}_{i}=\alpha_{3}+{{\beta_{3}}_{phoneme}}_{i}+{{\gamma_{3}}_{language}}_{i}+{{\delta_{3}}_{speaker}}_{i}+{\zeta_{3}}_{{language}_{i},{phoneme}_{i}}\\ +{\eta_{3}}_{{language}_{i},{speaker}_{i}}+{\theta_{3}}_{{speaker}_{i},{phoneme}_{i}}+{\iota_{3}}_{{language}_{i},{speaker}_{i},{phoneme}_{i}} \end{array}$$

Partial model equation for the formant model of speakers’ reference realizations of French and German vowels. See Appendix for full equation.

Here, too, the three formants are modeled as outcomes of a multivariate normal process. The means for the outcome distribution are an additive combination of the intercept α, the differences between the different phonemes (β), the differences between the languages French and German (γ), the differences between the speakers (δ), the two-way interactions between the three group-level effects *phoneme*, *language*, and *speaker* represented as ζ, η, and θ, respectively, in Equation 2, and their three-way interaction represented as ι. Like in the model displayed in Equation 1, all effects are themselves inferred from multivariate normal distributions, capturing the joint effects on all three formants simultaneously.

The second model is different from the first as it infers the formant values of all other vowels. This enables the contextualization of the results of Model 1 insofar as it shows how close their schwa realizations are to the realizations of all other vowels.

Model 2 contains the three formants of each vowel as dependent variables with *phoneme* (the vowel in question), *language* (the language of the realization: French vs. German), and *speaker* (which speaker produced the vowel) as predictors. We further included the two-way and three-way interactions between the predictors to capture potential interaction effects (e.g., if certain speakers have specifically high F1 values in the production of French [ø] than in German [ø]). This makes the model less interpretable; however, the purpose of Model 2 is to make accurate predictions for the formant values that can then be used as reference points for the interpretation of Model 1’s results. As in Model 1, all predictors of all vowels are jointly inferred as a multivariate varying intercept model to make more accurate predictions. The results from both models presented jointly in the following section. Model 2 analyzes the formant values of the L2/L1 realizations of various French and German vowels, while Model 1 infers the formant values of L2 schwa under different conditions (i.e., measuring the effects of different variables on those formants). For this reason, the following plots showing the results combine the posterior results of both Model 1 and Model 2.

## 3 Results

**Figure 1.**
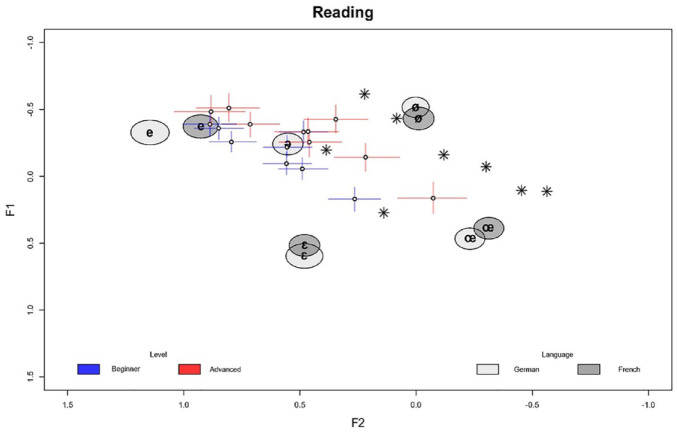
Reading task: normalized per-word F1/F2 estimates (crosses) in speakers by proficiency level along with reference realizations (ellipses) by language and native speaker’s target realizations (stars). Color version of the figure is available online.

**Figure 2.**
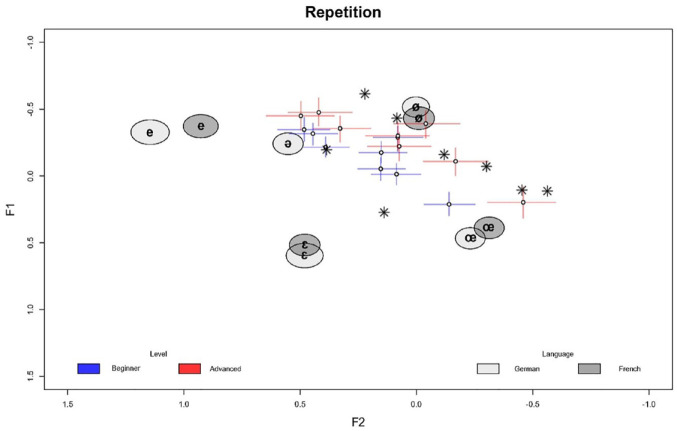
Repetition task: normalized per-word F1/F2 estimates (crosses) in speakers by proficiency level along with reference realizations (ellipses) by language and native speaker’s target realizations (stars). Color version of the figure is available online.

**Figure 3.**
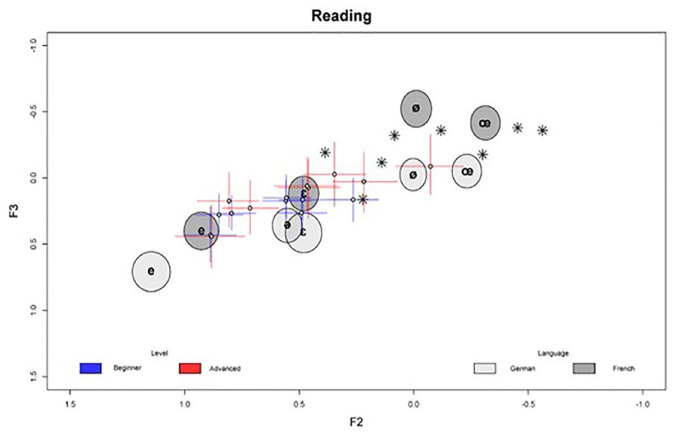
Reading task: normalized by-word F2/F3 estimates (crosses) in speakers by proficiency level along with reference realizations (ellipses) by language and native speaker’s target realizations (stars). Color version of the figure is available online.

**Figure 4.**
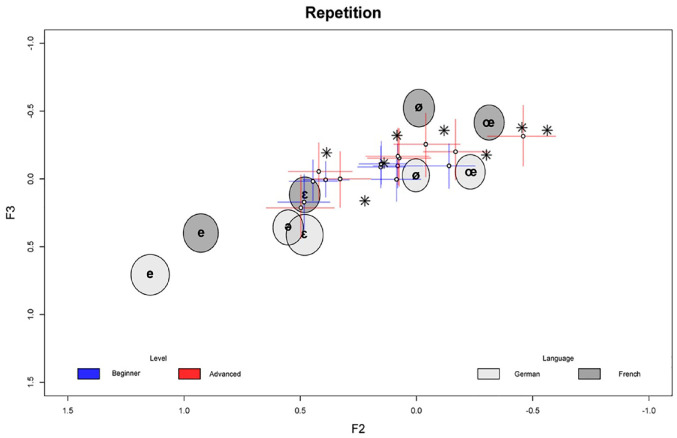
Repetition task: normalized by-word F2/F3 estimates (crosses) in speakers by proficiency level along with reference realizations (ellipses) by language and native speaker’s target realizations (stars). Color version of the figure is available online.

**Figure 5.**
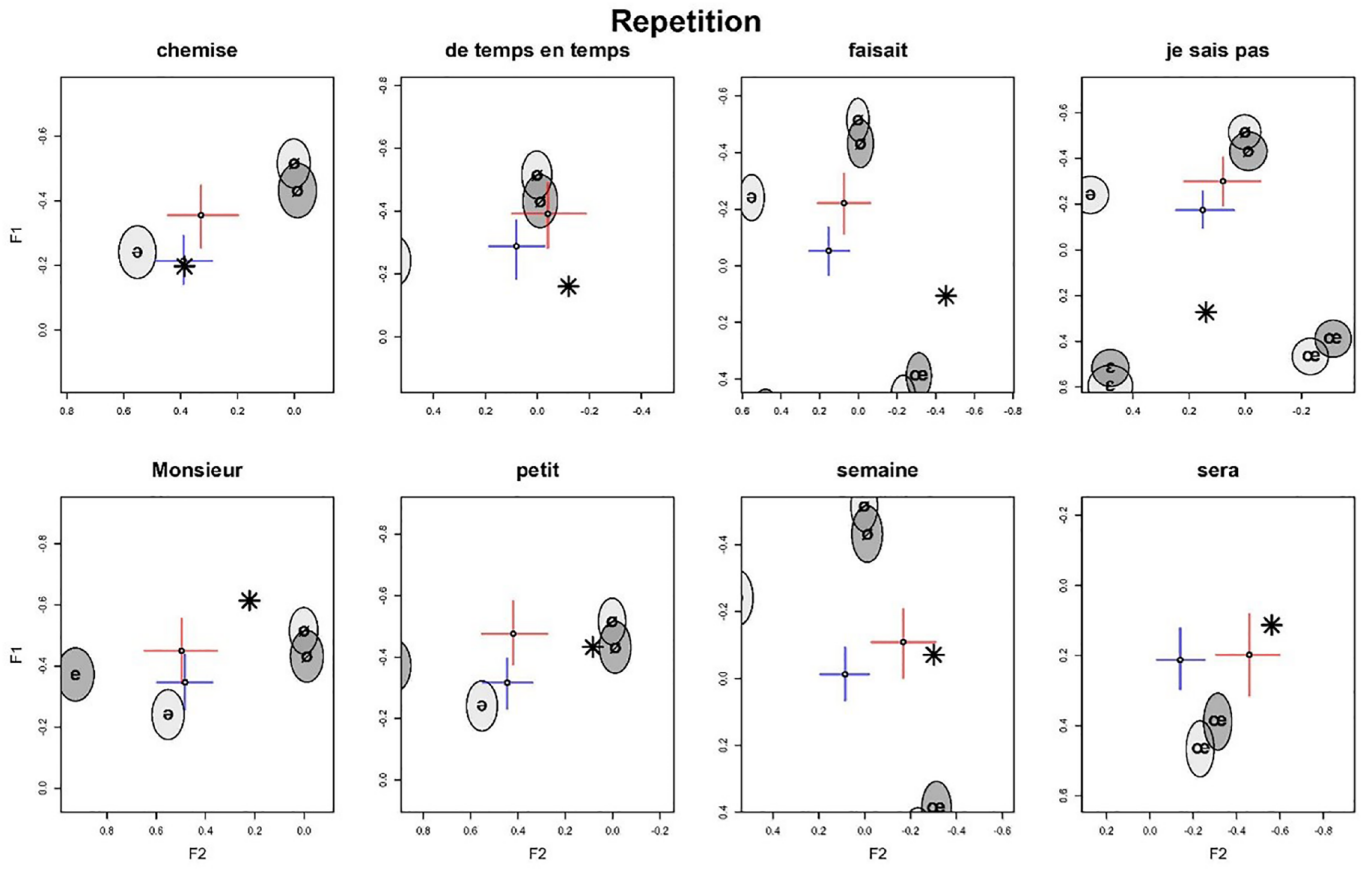
Repetition task: normalized by-word F1/F2 estimates (crosses) in speakers by proficiency level (blue = beginner, red = advanced) along with reference realizations (ellipses) by language and native speaker’s target realizations (stars). The plot shows the same results as Figure 2 with the difference that here, each word’s realization is displayed isolated and magnified. Because of this, the axis scales are different for every sub-plot. Color version of the figure is available online.

**Figure 6.**
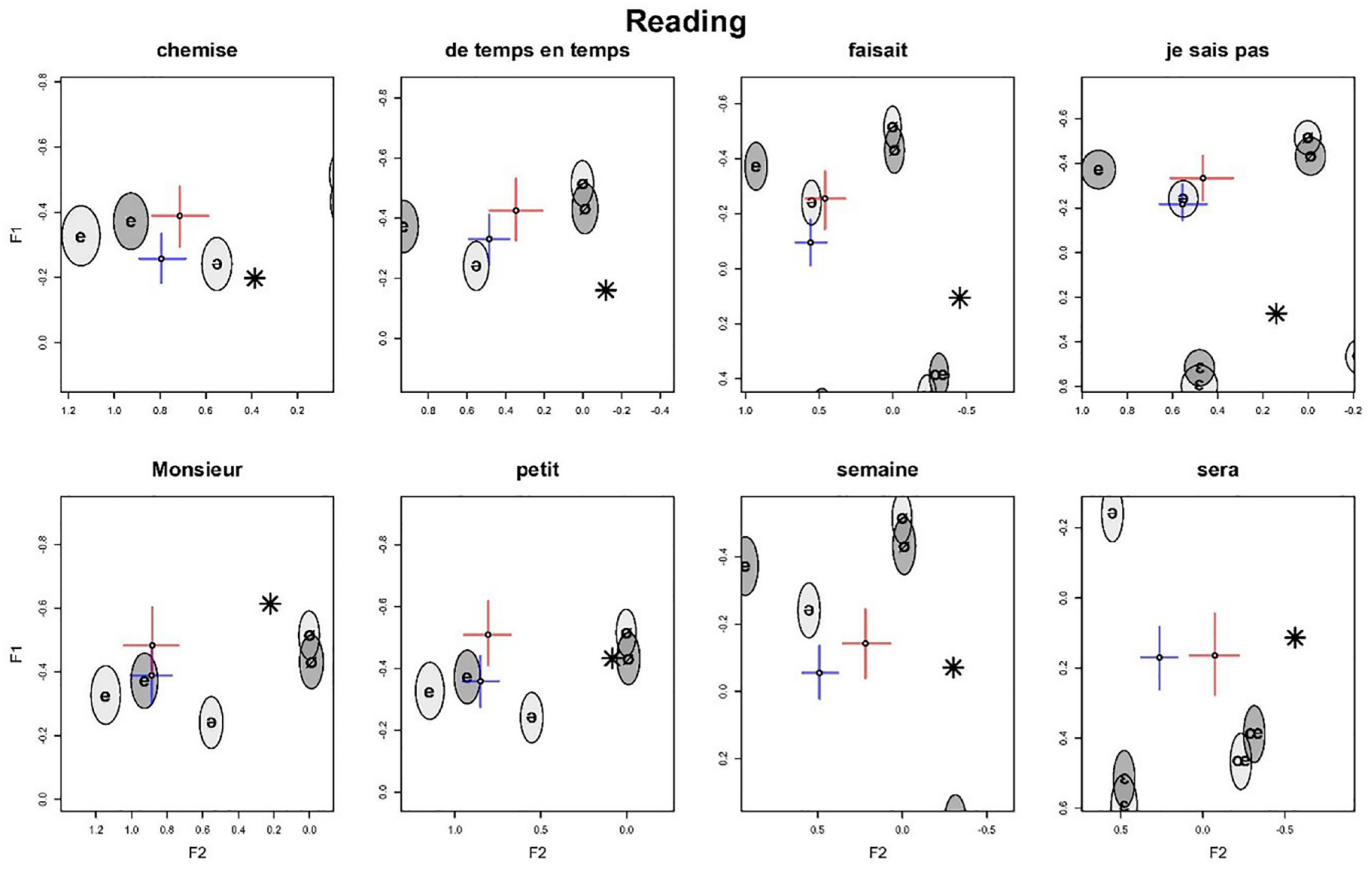
Reading task: normalized by-word F1/F2 estimates (crosses) in speakers by proficiency level (blue = beginner, red = advanced) along with reference realizations (ellipses) by language and native speaker’s target realizations (stars). The plot shows the same results as Figure 3 with the difference that here, each word’s realization is displayed isolated and magnified. Color version of the figure is available online.

In the following, we outline the model results in detail. Since the models are complex, we focus on the main findings while leaving small or inconclusive results aside. The full posterior summary of all model parameters is provided in the Appendix. Figures 1–6 show the posterior estimates^7^ for the model results concerning the realization of formant values of French schwa productions along with the inferred formant values of all other vowels for reference. In other words, the results from both models are plotted in the same plots, which is possible since both models, although operating with different predictors, have the three formants as outcomes and therefore make predictions about the same formant space. In all figures, the formants of the Schwa realizations are displayed as symbols (dots for the learners, stars for the native speakers’ reference productions) in F1 F2 space plotted on the x and y axes, respectively. The reference realizations (German and French vowels) produced by the reference model (see model in Section 2) are shown in gray ellipses. The shade of gray indicates the language of the speaker’s reference realization and the width of the ellipses (along both the x and y axes) show the 95% credible interval (CI). This means that the area of any ellipse covers 95% of the posterior draws (i.e., the true mean of the speaker’s realization for any given vowel has a 95% chance of falling into this ellipse). The per-word realization of schwa is indicated by the blue (beginner-level speakers) and red (advanced-level speakers) crosses, where the length of the segments indicates the 95% CI for each formant, and the center dot indicates the average of both formants’ posterior means. In practice, a cross shows the location of the (beginner or advanced) speaker’s schwa realization—hence, if two crosses or a cross and an ellipse overlap, their realizations are very close. The stars indicate the locations of the native speaker’s schwa realizations in the eight words. This being said, in Bayesian analysis, there is no significance criterion equivalent to that found in Frequentist contexts. Therefore, we focus on the degree of difference between the posterior estimates. As a rule of thumb, if two CIs do not overlap and the further they are apart, the more evidence there is for the two estimates to be distinct.

It needs to be kept in mind that all plotted items, with the exception of the native-speaker realizations (stars), are posterior model results merged from both models, which means that none of these figures contain summary statistics. In the figures, the mean expected locations of the phonetic realizations (ellipses) are obtained from Model 2, whereas the average expected formant locations of the L2 schwa realizations (crosses) are obtained through Model 1.

Figure 1 shows the formant estimates for the reading task. Recall that the formants were normalized before running the models (see Section 2). Thus, in the figures, the formants F1, F2, and F3 refer to the normalized formants.

We can observe in Figure 1 that the advanced-level group is generally closer to the native-speaker target realizations^8^ (stars) than the beginner-level group. We can observe this in the CIs, where the advanced groups are shifted in the negative F2 direction, and the overlap in this dimension is smaller. This effect is strongest for the two rightmost realizations. This indicates that there is a general effect of proficiency: with increased proficiency, French schwa realizations more closely align with the native-speaker realizations of schwa. This effect, moreover, is stronger for F2 than for F1 since most of the group differences between beginners and advanced learners can be attributed to F2. This is because the beginner and advanced estimates have a pronounced overlap along the F1 dimension. Furthermore, the group differences are strongest for words that have comparatively lower F2 values overall.

In general, we find that although the advanced-level realizations are more target-like, the realizations overall are clustered around the speaker’s native-language “schwa” realization in initial syllables (light gray ellipse). However, Figure 1 shows that in contrast to Standard German varieties, as expected (see Section 1), the Austrian students do not typically produce <e> in initial syllables as schwa, that is, as a central vowel, but more fronted, close to the unrounded front vowels [e] and [ε]. F1 values are similar to [e], whereas the associated F2 values instead resemble [ε]. Posterior CIs for these dimensions (ellipses) are very close to or even overlap with the respective realizations. Otherwise, in the reference realizations, there is considerable overlap between the realizations of the same vowels in German and French. The exception to this is the realization of [e], which shows a lower F2 for French than for German.

Figure 2 shows the normalized F1/F2 estimates for the repetition task. This is to compare Figures 1 and 2 to highlight differences and similarities between the tasks. In comparing both figures, we can see that the French schwa realizations of beginner-level learners are much closer to the native-speaker target realization in the repetition task than in the reading task, which results from the distribution of the posterior estimates being closer to the native-speaker realizations. This, in turn, means that there is a determinable difference between the two tasks in performance, with the realizations in the repetition task closer to the native-speaker target realizations.

To also look at F3, generally associated with lip rounding, in the learners’ productions of French schwa, Figure 3 shows the results for the formants F2 and F3 where we can observe that, similar to Figure 1, the group differences between beginner-level and advanced-level learners are most prominent in F2 with very little difference in F3. However, regarding the reference condition, it must be noted that “German schwa” pronounced by the 15- to 16-year-old Austrian high-school students is produced with considerably higher F3 values than [ø] and [œ], again indicating that German <e> in initial syllables is closer to the unrounded front vowels [e] and [ε] than to the rounded vowels [ø] and [œ].

The same is true for the formants F2 and F3 in the repetition task, where, in addition, the F2 values are generally lower, as already observed comparing Figures 1 and 2.

In addition to comparing the production of schwa not only between learner groups (beginners vs. intermediate) and tasks (orthography absent vs. present) but also between spelling conditions (<e> vs. letter combinations), Figure 5 presents a more targeted visualization of the results of the repetition task reported in Figure 2. Figure 5 shows separate panels for each word, with the axes adjusted to give a more magnified view of the word realization.

Figure 5 indicates that the differences between the beginner-level and advanced-level groups are strongest for the words *semaine* and *sera*, and at the same time, both are closer to the native-speaker target realization than the realizations in other words. Their CIs between beginner and advanced do not overlap; for *sera*, there is an even stronger difference between beginner and advanced in the F2 dimension. The learners are on-target with F1 in *chemise* (beginners only), *petit*, *semaine*, and *sera*, and they are on-target with F2 in *chemise*, *de temps en temps*, *je sais pas*, *semaine*, and *sera*. This can be seen in their posterior CIs which overlap with the target realizations in the particular dimension. Furthermore, the proficiency group difference in the individual words does not seem to be linked to the distance between the target realization and the beginner-level realization. In other words, even if a learner’s realization of schwa is very different from the native-speaker target in a particular word, there is little indication that the distance shrinks with increased proficiency.

In the reading task (see Figure 6) again, we can observe that *sera* and *semaine* show the strongest improvement of all the words, albeit with generally higher F2 values than the repetition task (see Figure 5). In general, none of the F2 realizations are on-target. Furthermore, the production patterns of French schwa in *Monsieur* and *petit* are also similar to those observed in the repetition task, and in that, in both cases, French schwa is produced similarly to unrounded closed front vowels by the beginner and advanced learners. However, in contrast to the imitation patterns of French schwa, in the reading task, French schwa in *chemise*, *de temps en temps*, *faisait*, and *je sais pas* is produced more fronted. Again, this is the case for both beginners and advanced learners. The difference in the *Task* variable concerns mainly the positioning of the realizations on the F2 axis, as realizations in the *Reading* task are shifted further in the positive F2 direction compared to the *Repetition* task.

**Figure 7.**
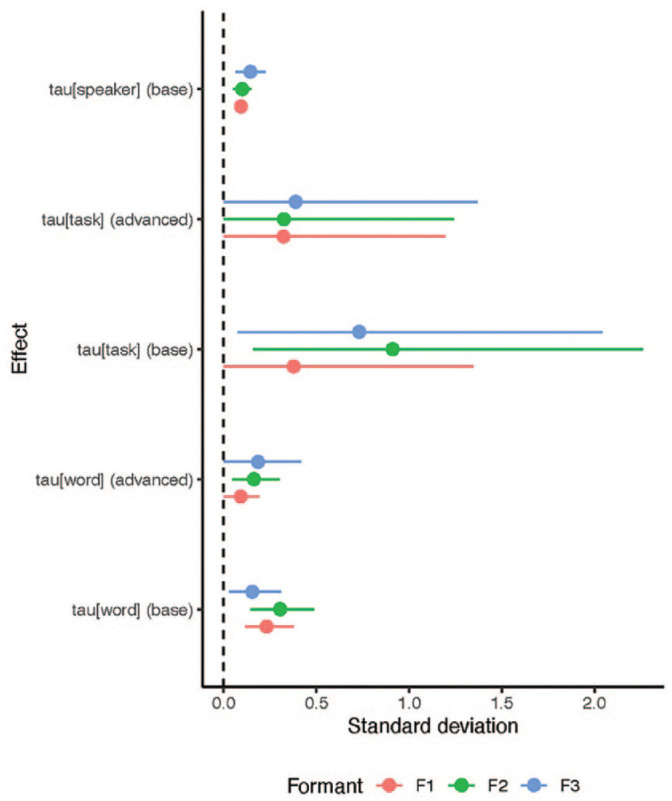
Posterior intervals (.95 Highest Density interval) of hierarchical variance parameters by predictor and base effect vs. effect of the advanced level of proficiency. The dashed vertical line indicates y = 0. Recall that, as per the model definition, the *speaker* group predictor is only calculated at the base effect level. Color version of the figure is available online.

Furthermore, we can analyze the posterior distributions of the hierarchical variance parameters (τ) that govern the standard deviation of the group-level varying effects (see Appendix), which gives insights into how heterogeneous the individual factor levels of the predictors (*speaker*, *task*, *word*) are. When the inferred standard deviation is high, it means that the factor levels of the predictor are more heterogeneous in their effects. For example, Figure 7 shows the posterior effects of each parameter by predictor (*speaker*, *task*, *word*) split up into effects of the beginner-level speakers (intercepts, marked as *base*) and the effects of the advanced level of proficiency (slopes, marked as *advanced*). These results are important for the analysis as the heterogeneity among the varying effects indicates how strongly the effects of the individual factor levels differ from each other. These results complement the previous results insofar as they are a macro-level perspective on the relationship between the factors *within* each variable rather than between the variables.

In Figure 7, we can observe that variance within the group clusters is generally low, while the variance of the *task* effect is very uncertain, indicated by large posterior CIs. This might be because there are only two tasks distinguished in the data which make the parameter difficult to infer. The most salient result that can be inferred with relative certainty is that the base effect of *word* shows higher heterogeneity, mostly for F1 and F2. Again, this means that the individual words are more different from one another than the differences between individual speakers. This means that the influence of the individual speaker is small, indicating that the findings presented in Figures 1–6 are robust across speakers.

Furthermore, we can analyze the inferred correlations between the three formants in each of the group-level parameters (*word*, *task*, *speaker*). Doing this gives us insight into how the three formants are correlated across all conditions. We can thus show that some formants are correlated with respect to speaker proficiency level. This again helps to contextualize the findings presented in Figures 1–6 from a macro-level perspective.

**Figure 8.**
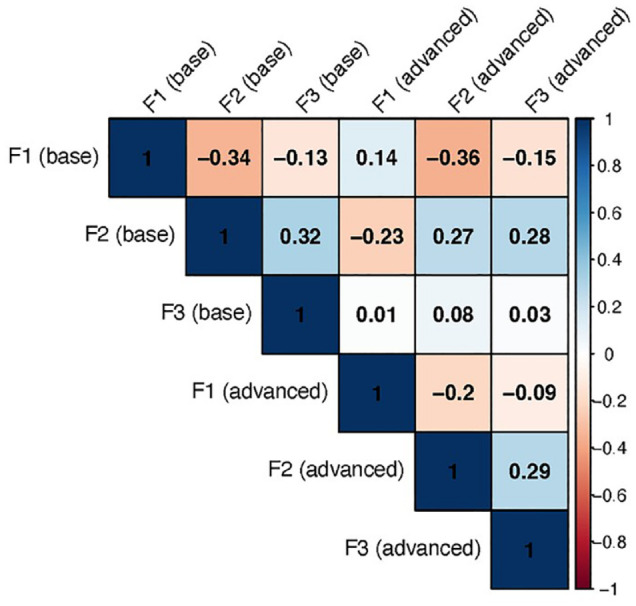
Plot of the posterior mean correlations (variable *R*, see Appendix) between the three formants in the group-level effect of *word*, split up by base effect vs. effect of advanced-level proficiency. The color and figure in the squares indicate the magnitude of the correlation. Color version of the figure is available online.

We find that only *word* shows correlations with low uncertainty and magnitudes of>.3, which we consider to be notable. Figure 8 shows a correlation plot of these parameters.

In this figure, we can see that there are several medium-strong correlations between the formants. First, the base effects of F1 and F2 correlate negatively, which indicates that on the word level, words with higher F1 values tend to have lower F2 values and vice versa. A similar situation in the positive direction exists between the base effects of F2 and F3. This shows that all three effects are linked on the word level, which means that for individual words, the formant realizations are interdependent, which is expected. Similarly, the advanced-level effects (see Figure 8) show some correlation with other effects as well with, for example, the advanced-level parameter of F2 is negatively correlated with the base level of F1. In other words, in words with higher F1 base values, the language proficiency effect on F2 is lower and vice versa. The interpretation of these values is that the advanced-level group shows that F1 and F2 are correlated even across groups.

## 4 Discussion

The present study investigated the potential influence of orthography related to L2 phonolexical representations by examining imitation and production patterns of a perceptually easy L2 phone with incongruent grapheme-phoneme correspondences between the L1 and L2. Specifically, two groups of Austrian high-school students (31 beginners and 19 intermediate learners) were tested on a word-repetition and a word-reading task for the production of French schwa in initial syllables (*semaine* ([s[mεn] “week”]—acoustically and articulatorily close to the French full-front rounded vowels [œ] and [ø]). Crucially, French words were chosen to be either spelled such that schwa was represented by the letter <e> or by other letter combinations such as <on> or <ai> . Formant values (F1 F3) of the learners’ schwa productions for each task were extracted and used, first, to examine the differences between production patterns of the two groups of learners within each task and, second, to assess the relationship between L1 and L2 categories. For the latter, formant values of the learners’ schwa productions were compared to those of French and German full vowels [œ], [øː], [e], and [ε], as well as to German schwa, recorded from a subset of the learners in a reading task (see Section 2). To our knowledge, L2 categories that exist in the L1 (e.g., Germ. *Löffel* [ˈlœfl̩] “spoon,” *Flöte* [ˈfløːtə] “flute”) but do not overlap in terms of grapheme-phoneme correspondences have not been considered in previous research but could widen our knowledge about the sources of L2 phonolexical representations and the mapping between them. Although the findings of the present study should be interpreted with caution as they are based on only one L2 category examined in a limited collection of L2 words produced by a particular group of learners, they represent a first step toward a better characterization of the phonology/orthography interface in L2 phonolexical representations, which will be discussed in more detail below.

Before examining French schwa realizations in greater detail, a preliminary analysis was conducted to assess pronunciation accuracy of perceptually easy L2 vowels with (largely) congruent grapheme-phoneme correspondences between the L1 and L2 ([ε], [e], [œ], [ø]) read aloud by Austrian learners of French. An analysis of the first three formants showed that production patterns of the French and German front vowels strongly overlapped. These results replicated findings of previous studies suggesting that L2 categories that also exist in the L1 do not pose any problems for learners (e.g., Broersma, 2005; Escudero et al., 2014; Flege, 1987; Llompart & Reinisch, 2018; Weber & Cutler, 2004). Another relevant finding was that, as expected, German <e> in initial unstressed syllables pronounced by Austrian students was more fronted and less rounded, that is, similar to [e̞], rather than realized as German schwa as could have been expected for Standard German varieties (Hahn & Siebenhaar, 2017; Krech et al., 2009; Moosmüller et al., 2015).

The main question was, however, whether French schwa in initial syllables was mapped onto the phonetically closest L1 categories or onto L1 grapheme-phoneme correspondences. If the former was the case, Austrian German learners could be expected to produce all French schwas as tokens of [œ] or [øː], and therefore target-like (see Section 1). If the latter was the case, French schwa productions by the learners were expected to be close to German <e> realizations, that is, close to the unrounded front vowels [ε], [e], and [e̞]. This was assessed by comparing imitation and production patterns of French words, including schwa in initial syllables. The results showed that French schwa realizations were error-prone in both word repetition and word reading, in that mispronunciations were present regardless of whether orthographic input was present or not. However, the Bayesian hierarchical regression model with multivariate normal outcomes for the first three formants (see Section 2) revealed that there was a determinable effect of *task* on performance with the realizations in the repetition task being closer to the rounded front vowels [œ] or [øː], and therefore more target-like than in the word-reading task. In the latter, the Austrian students tended to produce French with higher F2 values, that is, similar to closed unrounded front vowels [e] and [e̞]. This is, in principle, not surprising, as our results replicate findings of previous studies outlining that imitating difficult L2 contrasts is less challenging for learners than producing them without an auditory input (e.g., Hao & de Jong, 2016; Llompart & Reinisch, 2018; Rojczyk, 2013; Rojczyk et al., 2013). Given that we not only found noticeable differences between formant values of learners’ schwa production in the word-repetition and word-reading tasks but also between the learners’ schwa imitation patterns and the native speakers’ auditory-acoustic patterns, especially along the back-front axis (F2), contrary to the suggestions of previous studies (e.g., Hao & de Jong, 2016; Rojczyk, 2013; Rojczyk et al., 2013), the apparent discrepancy between the two tasks cannot be interpreted as bypassing of phonological processes during imitation. Rather, our findings provide further evidence that imitation patterns seem to be related to phonolexical representations (e.g., Flege & Eefting, 1988; Jia et al., 2006; Llompart & Reinisch, 2018; Schouten, 1977).

This assumption is supported by the fact that there are clear differences between the words in the imitation of French schwa in that some words were more error-prone than others: while schwa was imitated more or less target-like in *chemise*, *de temps en temps*, *je sais pas*, *faisait*, *semaine*, and *sera*, schwa in *petit* and *Monsieur* was produced more fronted, that is, close to [e] and [e̞]. These findings indicate that spelling conditions, that is, whether schwa corresponds to <e> (*chemise*, *semaine*, *sera*, *petit*, *je sais pas*, *de temps en temps*) or other letter combinations, such as <on> (*Monsieur*) and <ai> (*faisait*), seem not to be predictive of imitation patterns of French schwa. In word reading, by contrast, the students tended to produce French schwa in all words similar to the closed unrounded front vowels [e] and [e̞]. However, this was not only the case for schwa corresponding to <e> but also for schwa in *faisait*, which would have been pronounced as [aɪ̯] if it is pronounced according to the L1 grapheme-phoneme correspondences. Realization patterns of [e] and [e̞] might be explained by the fact that in French, the digraph <ai> is commonly pronounced as unrounded front vowel, for example, *faire* [fεʁ] “to make,” *fait* [fε] “(he or she) makes,” *fais* [fε] “(I/you) make.” In open syllable, the realization of <ai> can vary in terms of openness. This indicates that not only do L1 grapheme-phoneme correspondences play in role L2 reading but also the frequency of L2 grapheme-phoneme correspondences. A potential explanation for the tendency of mispronunciation of schwa in *petit* and *Monsieur* in both tasks is that both are highly frequently used words, which are even used in the L1 (Germ. *Monsieur* [me̞sjø], *Petits-Fours* [pe̞tiˈfuːɐ̯]). It might be that such words are most likely to be heard as *[petit] or *[məsjø] in the classroom, while words such as *sera* or *faisait* are less frequent in classroom settings and therefore also less likely to be heard as mispronounced. As Llompart (2021a) has suggested for (non-)word rejection, these results indicate an effect of exposure to native and non-native input on the encoding of L2 phonological categories in that students fail to fully generalize the schwa realization and apply it predominantly in known contexts where high-frequency exemplars exist. Such effects can be compounded if this underlying disparity in target-likeness between words is passed on to the students by teachers who are themselves non-native speakers.

This hypothesis is strengthened by the fact that the most salient effect that can be determined with relative certainty in our Bayesian hierarchical regression model is not the base effect of *task* or *level of proficiency*, but the group effect of *word*. This means that any effect such as L2 proficiency or reading vs. repetition does not act on every word uniformly. This provides further evidence that the ability to encode L2 categories into representations of words in the L2 lexicon does not automatically improve with increased proficiency (e.g., Llompart, 2021). The proficiency group difference in the individual words does not seem to be linked to the distance between the target realization and the beginner-level realization: Even if a learner’s realization of schwa is very different from the native-speaker target in a particular word, there is little indication that the distance shrinks with increased proficiency. In other words, for words in which schwa realizations are associated with high F2 values, very little difference between the two groups can be observed. This indicates that French schwa pronunciation in certain words poses fewer problems for Austrian learners from the beginning, while in other words, schwa is mispronounced even at a higher proficiency level in word repetition as well as in word reading, albeit F2 values are generally lower in the repetition task. Interestingly, our results show that F1 and F2 are correlated even across groups. This means, there is a high chance that students that tend to pronounce schwa correctly in the beginning produce schwa also correctly in the sixth year of learning, while students that have difficulties in the beginning also have problems in the sixth of learning. This indicates that in the case of the Austrian learners, pronunciation patterns of French schwa do not necessarily evolve along different proficiency levels exclusively, indicating that some words’ schwa realizations are fossilized (or subject to only very little change) while we can see progress in other words. However, note that this is not a longitudinal study, and therefore only an assumption that needs to be tested in further research on longitudinal data.

In sum, the present study provides new insights into the encoding of phonolexical representation in the light of L2 production data. By comparing imitation and production patterns of a perceptually easy L2 phone with incongruent phoneme-grapheme correspondences between the L1 and L2, it gives further approximations to the question of how phonology and orthography interact during phonolexical encoding. Against the shared claim of most L2 phonology learning models (Best & Tyler, 2007; Flege, 1995; Flege & Bohn, 2021; van Leussen & Escudero, 2015) and previous studies on the development of L2 phonolexical representations (e.g., Gor et al., 2021, Llompart, 2021a, 2021b) suggesting that establishing a new L2 phonological category that is phonetically close to an L1 category is relatively easy for foreign-language learners, this study provides evidence that acoustic and articulatory proximity does not appear to be the only factor in creating L2 categories of perceptually easy phones. Imitation and production data suggest that there seems to be a complex interplay of different factors that lead to success or failure in L2 phonolexical encoding. In the case of incongruent grapheme-phoneme correspondences between the L1 and L2, especially when orthographic input is present, orthography appears to outweigh phonetic proximity between L1 and L2 categories (e.g., Bassetti, 2007, 2023; Bassetti & Atkinson, 2015; Hayes-Harb & Cheng, 2016; Hayes-Harb et al., 2010; Mathieu, 2016). Hence, orthography seems to be an additional challenge for L2 phonolexical encoding and therefore another source for “fuzziness” in L2 lexical representations. However, orthography does not appear to play the dominant role either, as learners do not map French schwa systematically onto L1 grapheme-phoneme correspondences. The results provide evidence that L2 development patterns are highly determined by speaker and especially lexical item. One reason for this might be L1 and L2 (error-prone) oral input (as mentioned above) and the amount of explicit pronunciation training or lack thereof. We therefore argue that L2 phonetic and phonological development depends not only on the proximity between L1 and L2 phones but also on a highly complex interaction of multiple internal and external factors beyond orthography and phonology. However, in the absence of data about the quantity and quality of the students’ input (i.e., production patterns of schwa in initial syllables by the teachers), the audio material used in the classroom, and the amount of pronunciation training, this must be interpreted with caution and should be investigated in further research. Despite its limitations, the present study presents a first step for upcoming research in this field. Future studies may assess production patterns of other L1 categories produced by learners with a different L1 (Barrios & Hayes-Harb, 2021) and use experimental paradigms testing word recognition and lexical decision for (non-)words. They may also include perceptually and articulatorily easy L2 sounds with incongruent grapheme-phoneme correspondences between the L1 and L2.

## Appendix

$$\begin{array}{c} \left[{F_{1}}_{i},{F_{2}}_{i},{F_{3}}_{i}]\sim MultiNormal([{\mu_{1}}_{i},{\mu_{2}}_{i},{\mu_{3}}_{i}],K\right)\\ {\mu_{1}}_{i}=\alpha_{1}+{{\beta_{1}}_{task}}_{i}+{{\gamma_{1}}_{word}}_{i}+{{\delta_{1}}_{speaker}}_{i}\\ +(\epsilon_{1}+{{\zeta_{1}}_{task}}_{i}+{{\eta_{1}}_{word}}_{i})L_{i}\\ {\mu_{2}}_{i}=\alpha_{2}+{{\beta_{2}}_{task}}_{i}+{{\gamma_{2}}_{word}}_{i}+{{\delta_{2}}_{speaker}}_{i}\\ +(\epsilon_{2}+{{\zeta_{2}}_{task}}_{i}+{{\eta_{2}}_{word}}_{i})L_{i}\\ {\mu_{3}}_{i}=\alpha_{3}+{{\beta_{3}}_{task}}_{i}+{{\gamma_{3}}_{word}}_{i}+{{\delta_{3}}_{speaker}}_{i}\\ +(\epsilon_{3}+{{\zeta_{3}}_{task}}_{i}+{{\eta_{3}}_{word}}_{i})L_{i}\\ \left[\alpha_{1},\ldots ,\alpha_{3},\epsilon_{1},\ldots ,\epsilon_{3}]\sim MultiNormal([{\bar{\mu }}_{1},\ldots ,{\bar{\mu }}_{6}],K_{b}\right)\\ \left[{{\beta_{1}}_{task}}_{i},\ldots ,{{\beta_{3}}_{task}}_{i},{{\zeta_{1}}_{task}}_{i},\ldots ,{{\zeta_{3}}_{task}}_{i}\sim MultiNormal([0,\ldots ,0]K_{t})\right]\\ \left[{{\gamma_{1}}_{word}}_{i},\ldots ,{{\gamma_{3}}_{word}}_{i},{{\eta_{1}}_{word}}_{i},\ldots ,{{\eta_{3}}_{word}}_{i}]\sim MultiNormal([0,\ldots ,0]K_{w}\right)\\ \left[{{\delta_{1}}_{speaker}}_{i},\ldots ,{{\delta_{3}}_{speaker}}_{i}]\sim MultiNormal([0,\ldots ,0]K_{s}\right)\\ K=(\begin{array}{ccc} \tau_{1} & \cdots & 0\\ \vdots & \ddots & \vdots \\ 0 & \cdots & \tau_{3} \end{array})R(\begin{array}{ccc} \tau_{1} & \cdots & 0\\ \vdots & \ddots & \vdots \\ 0 & \cdots & \tau_{3} \end{array})\\ K_{b}=(\begin{array}{ccc} {\tau_{b}}_{1} & \cdots & 0\\ \vdots & \ddots & \vdots \\ 0 & \cdots & {\tau_{b}}_{6} \end{array})R_{b}(\begin{array}{ccc} {\tau_{b}}_{1} & \cdots & 0\\ \vdots & \ddots & \vdots \\ 0 & \cdots & {\tau_{b}}_{6} \end{array})\\ K_{t}=(\begin{array}{ccc} {\tau_{t}}_{1} & \cdots & 0\\ \vdots & \ddots & \vdots \\ 0 & \cdots & {\tau_{t}}_{6} \end{array})R_{t}(\begin{array}{ccc} {\tau_{t}}_{1} & \cdots & 0\\ \vdots & \ddots & \vdots \\ 0 & \cdots & {\tau_{t}}_{6} \end{array})\\ K_{w}=(\begin{array}{ccc} {\tau_{w}}_{1} & \cdots & 0\\ \vdots & \ddots & \vdots \\ 0 & \cdots & {\tau_{w}}_{6} \end{array})R_{w}(\begin{array}{ccc} {\tau_{w}}_{1} & \cdots & 0\\ \vdots & \ddots & \vdots \\ 0 & \cdots & {\tau_{w}}_{6} \end{array})\\ K_{s}=(\begin{array}{ccc} {\tau_{s}}_{1} & \cdots & 0\\ \vdots & \ddots & \vdots \\ 0 & \cdots & {\tau_{s}}_{3} \end{array})R_{s}(\begin{array}{ccc} {\tau_{s}}_{1} & \cdots & 0\\ \vdots & \ddots & \vdots \\ 0 & \cdots & {\tau_{s}}_{3} \end{array})\\ {\bar{\mu }}_{1},\ldots ,{\bar{\mu }}_{6}\sim Normal(0,0.5)\\ R,R_{b},R_{t},R_{w},R_{s}\sim LKJ(2)\\ \tau ,\tau_{t},\tau_{w},\tau_{s}\sim Exponential(1)\\ \tau_{b}\sim Exponential(2) \end{array}$$

$$\begin{array}{c} \left[{F_{1}}_{i},{F_{2}}_{i},{F_{3}}_{i}]MultiNormal([{\mu_{1}}_{i},{\mu_{2}}_{i},{\mu_{3}}_{i}],K\right)\\ {\mu_{1}}_{i}=\alpha_{1}+{{\beta_{1}}_{phoneme}}_{i}+{{\gamma_{1}}_{language}}_{i}+{{\delta_{1}}_{speaker}}_{i}+{\zeta_{1}}_{{language}_{i},{phoneme}_{i}}\\ +{\eta_{1}}_{{language}_{i},{speaker}_{i}}+{\theta_{1}}_{{speaker}_{i},{phoneme}_{i}}+{\iota_{1}}_{{language}_{i},{speaker}_{i},{phoneme}_{i}}\\ {\mu_{2}}_{i}=\alpha_{2}+{{\beta_{2}}_{phoneme}}_{i}+{{\gamma_{2}}_{language}}_{i}+{{\delta_{2}}_{speaker}}_{i}+{\zeta_{2}}_{{language}_{i},{phoneme}_{i}}\\ +{\eta_{2}}_{{language}_{i},{speaker}_{i}}+{\theta_{2}}_{{speaker}_{i},{phoneme}_{i}}+{\iota_{2}}_{{language}_{i},{speaker}_{i},{phoneme}_{i}}\\ {\mu_{3}}_{i}=\alpha_{3}+{{\beta_{3}}_{phoneme}}_{i}+{{\gamma_{3}}_{language}}_{i}+{{\delta_{3}}_{speaker}}_{i}+{\zeta_{3}}_{{language}_{i},{phoneme}_{i}}\\ +{\eta_{3}}_{{language}_{i},{speaker}_{i}}+{\theta_{3}}_{{speaker}_{i},{phoneme}_{i}}+{\iota_{3}}_{{language}_{i},{speaker}_{i},{phoneme}_{i}}\\ \left[\alpha_{1},\ldots ,\alpha_{3}]\sim MultiNormal([{\bar{\mu }}_{1},\ldots ,{\bar{\mu }}_{6}],K_{b}\right)\\ \left[{{\beta_{1}}_{phoneme}}_{i},\ldots ,{{\beta_{3}}_{phoneme}}_{i}]\sim MultiNormal([0,\ldots ,0]K_{p}\right)\\ \left[{{\gamma_{1}}_{language}}_{i},\ldots ,{{\gamma_{3}}_{language}}_{i}]\sim MultiNormal([0,\ldots ,0]K_{l}\right)\\ \left[{{\delta_{1}}_{speaker}}_{i},\ldots ,{{\delta_{3}}_{speaker}}_{i}]\sim MultiNormal([0,\ldots ,0]K_{s}\right)\\ \left[{\zeta_{1}}_{{language}_{i},{phoneme}_{i}},\ldots ,{\zeta_{3}}_{{language}_{i},{phoneme}_{i}}]\sim MultiNormal([0,\ldots ,0]K_{lp}\right)\\ \left[{\eta_{1}}_{{language}_{i},{speaker}_{i}},\ldots ,{\eta_{3}}_{{language}_{i},{speaker}_{i}}]\sim MultiNormal([0,\ldots ,0]K_{ls}\right)\\ \left[{\theta_{1}}_{{speaker}_{i},{phoneme}_{i}},\ldots ,{\theta_{3}}_{{speaker}_{i},{phoneme}_{i}}]\sim MultiNormal([0,\ldots ,0]K_{sp}\right)\\ \left[{\delta_{1}}_{{language}_{i},{speaker}_{i},{phoneme}_{i}},\ldots ,{\delta_{3}}_{{language}_{i},{speaker}_{i},{phoneme}_{i}}]\sim MultiNormal([0,\ldots ,0]K_{lsp}\right)\\ K=(\begin{array}{ccc} \tau_{1} & \cdots & 0\\ \vdots & \ddots & \vdots \\ 0 & \cdots & \tau_{3} \end{array})R(\begin{array}{ccc} \tau_{1} & \cdots & 0\\ \vdots & \ddots & \vdots \\ 0 & \cdots & \tau_{3} \end{array})\\ K_{b}=(\begin{array}{ccc} {\tau_{b}}_{1} & \cdots & 0\\ \vdots & \ddots & \vdots \\ 0 & \cdots & {\tau_{b}}_{3} \end{array})R_{b}(\begin{array}{ccc} {\tau_{b}}_{1} & \cdots & 0\\ \vdots & \ddots & \vdots \\ 0 & \cdots & {\tau_{b}}_{3} \end{array})\\ K_{p}=(\begin{array}{ccc} {\tau_{p}}_{1} & \cdots & 0\\ \vdots & \ddots & \vdots \\ 0 & \cdots & {\tau_{p}}_{3} \end{array})R_{p}(\begin{array}{ccc} {\tau_{p}}_{1} & \cdots & 0\\ \vdots & \ddots & \vdots \\ 0 & \cdots & {\tau_{p}}_{3} \end{array})\\ K_{l}=(\begin{array}{ccc} {\tau_{l}}_{1} & \cdots & 0\\ \vdots & \ddots & \vdots \\ 0 & \cdots & {\tau_{l}}_{3} \end{array})R_{l}(\begin{array}{ccc} {\tau_{l}}_{1} & \cdots & 0\\ \vdots & \ddots & \vdots \\ 0 & \cdots & {\tau_{l}}_{3} \end{array})\\ K_{s}=(\begin{array}{ccc} {\tau_{s}}_{1} & \cdots & 0\\ \vdots & \ddots & \vdots \\ 0 & \cdots & {\tau_{s}}_{3} \end{array})R_{s}(\begin{array}{ccc} {\tau_{s}}_{1} & \cdots & 0\\ \vdots & \ddots & \vdots \\ 0 & \cdots & {\tau_{s}}_{3} \end{array})\\ K_{\mathrm{lp}}=(\begin{array}{ccc} {\tau_{lp}}_{1} & \cdots & 0\\ \vdots & \ddots & \vdots \\ 0 & \cdots & {\tau_{lp}}_{3} \end{array})R_{lp}(\begin{array}{ccc} {\tau_{lp}}_{1} & \cdots & 0\\ \vdots & \ddots & \vdots \\ 0 & \cdots & {\tau_{lp}}_{3} \end{array})\\ K_{\mathrm{ls}}=(\begin{array}{ccc} {\tau_{ls}}_{1} & \cdots & 0\\ \vdots & \ddots & \vdots \\ 0 & \cdots & {\tau_{ls}}_{3} \end{array})R_{ls}(\begin{array}{ccc} {\tau_{ls}}_{1} & \cdots & 0\\ \vdots & \ddots & \vdots \\ 0 & \cdots & {\tau_{ls}}_{3} \end{array})\\ K_{\mathrm{sp}}=(\begin{array}{ccc} {\tau_{sp}}_{1} & \cdots & 0\\ \vdots & \ddots & \vdots \\ 0 & \cdots & {\tau_{sp}}_{3} \end{array})R_{sp}(\begin{array}{ccc} {\tau_{sp}}_{1} & \cdots & 0\\ \vdots & \ddots & \vdots \\ 0 & \cdots & {\tau_{sp}}_{3} \end{array})\\ K_{\mathrm{lsp}}=(\begin{array}{ccc} {\tau_{lsp}}_{1} & \cdots & 0\\ \vdots & \ddots & \vdots \\ 0 & \cdots & {\tau_{lsp}}_{3} \end{array})R_{lsp}(\begin{array}{ccc} {\tau_{lsp}}_{1} & \cdots & 0\\ \vdots & \ddots & \vdots \\ 0 & \cdots & {\tau_{lsp}}_{3} \end{array})\\ {\bar{\mu }}_{1},\ldots ,{\bar{\mu }}_{3}\sim Normal(0,1)\\ R,R_{b},R_{p},R_{l},R_{s},R_{lp},R_{ls},R_{sp},R_{lsp}\sim LKJ(2)\\ \tau ,\tau_{b},\tau_{p},\tau_{l},\tau_{s},\tau_{lp},\tau_{ls},\tau_{sp},\tau_{lsp}\sim Exponential(1) \end{array}$$

## Notes

1. There are also other types of L1 L2 relations described in the models but not discussed here.

2. Strictly speaking, French was their third or fourth language—all participants were already learning English as their first foreign language, and some of them were raised bilingual. French was consequently their second foreign language. However, for simplicity, we refer to French as the L2, signifying that it is a foreign language learned in a community where French is not spoken by the majority.

3. In certain cases, French schwa can alternate with zero within the same lexical context (Fougeron et al., 2007, e.g., *semaine* [səˈmεn] or [smεn]), and its alternation is strongly influenced by linguistic and extralinguistic factors (Armstrong, 2001; Lyche, 2016). When preceded by a pause, as is the case in a word list, schwa in first syllables of polysyllables is traditionally described as obligatory; more recent research, however, describes regional and lexical variation in this phonotactic context (Hansen, 1994; Racine, 2008).

4. However, in spontaneous (informal) speech, usually in this construction, not only the *ne* is omitted, but the construction can be also pronounced as [ʃepa] (Wachs, 1997).

5. The model objects, the code, and the output are published on Open Science Forum (OSF: https://osf.io/v2kwx/) DOI 10.17605/OSF.IO/V2KWX

6. A multivariate normal distribution is a probability distribution which is a multi-dimensional Gaussian distribution with means of size 3 (in this case) and a covariance matrix of size 3 × 3, which captures the covariances between the formants. This makes it possible to model all three formants and the predictors in the same model as opposed to running three separate models.

7. In Bayesian statistics, the posterior distribution is the probability distribution of a given model parameter (e.g., a predictor) after the model has run. Every parameter has such a posterior distribution, and it represents the uncertainty in the parameter’s strength. These posterior distributions can be summarized using CIs, which means that a 95% CI of, for example, –1 to 1, for a hypothetical parameter means that there is a 95% chance that the “true” value falls within this interval. Larger intervals mean that there is more uncertainty in the parameter estimate while narrower intervals mean more certainty about a parameter’s value.

8. Since the quality of French schwa seems to vary greatly, not only due to regional and inter-speaker differences but also depending on the surrounding segmental context (Bürki et al., 2008; Fougeron et al., 2007), it is not surprising that the formant values of the French schwa productions realized by the native speaker also vary across words.
